# CENPO regulated proliferation and apoptosis of colorectal cancer in a p53-dependent manner

**DOI:** 10.1007/s12672-022-00469-2

**Published:** 2022-02-03

**Authors:** Zhicheng Liu, Chuangqi Chen, Mei Yan, Xiangtai Zeng, Yuchao Zhang, Dongming Lai

**Affiliations:** 1 Department of Gastrointestinal Surgery, Sun Yat-sen Memorial Hospital of Sun Yat-sen University, 107 Yanjiang West Road, Guangzhou, 510120 Guangdong Province China; 2 Department of Gastrointestinal Surgery, The First Hospital of Jilin University, 71 Xinmin Street, Changchun, Jilin China; 3 Department of Colorectal Surgery, Center of Gastrointestinal Surgery, The First Affiliated Hospital of Sun Yat-sen University, 58 2nd Zhongshan Road, Guangzhou, Guangdong Province China; 4 Department of The First Affiliated Hospital, GanNan Medical University, 23 Qingnian Road, Ganzhou, Jiangxi China

**Keywords:** CRC, CENPO, Proliferation, Apoptosis, Migration

## Abstract

Colorectal cancer (CRC) is considered to be a leading cause of cancer-related death. Centromere protein O (CENPO) can prevent the separation of sister chromatids and cell death after spindle injury. Nevertheless, the role of CENPO in CRC has not been reported. The expression level of CENPO in CRC was revealed by TCGA database and immunohistochemical (IHC) staining. Subsequently, the loss-of-function assays were performed to identified the role of CENPO in CRC in vitro and in vivo. Our data demonstrated that CENPO was highly expressed in CRC. The expression of CENPO was positively correlated with the deterioration of CRC. Moreover, CENPO knockdown inhibited the malignant phenotypes of CRC cells, which was characterized by slowed proliferation, cycle repression at G2, promotion of apoptosis, reduced migration and weakened tumorigenesis. Furthermore, CENPO knockdown downregulated the expression of N-cadherin, Vimentin, Snail, CCND1, PIK3CA and inhibited AKT phosphorylation in CRC cells. Moreover, the function of CENPO in regulating proliferation and apoptosis depended on p53. In summary, CENPO may play a promoting role in CRC through the epithelial mesenchymal transition (EMT) and PI3K/AKT signaling pathway, which can be regarded as a molecular therapeutic target for CRC.

**Supplementary Information:**

The online version contains supplementary material available at 10.1007/s12672-022-00469-2.

## Introduction

Colorectal cancer (CRC) is the third most common cancer in the world, and the proportion of women is slightly higher than that of men [1]. As with other types of cancer, mutations in certain genes, such as oncogenes and tumor suppressor genes, may contribute to the development of CRC [2]. CRC can be divided into sporadic, hereditary and familial based on the different pathways of mutations such as chromosomal instability (CIN), microsatellite instability (MSI) and CpG island methylation phenotype (CIMP) [3–5]. At present, treatment options for CRC patients involve multimodal treatment, such as surgery and radiotherapy [6]. Considering the limitations of surgery and the side effects of chemotherapy such as low selectivity and systemic toxicity [7], immunotherapy and targeted therapy have entered clinical application. In addition, there has been significant progress in targeted therapies, such as bevacizumab, aflibercept, regorafenib, cetuximab and panitumumab have been approved for the treatment of metastatic CRC [8]. Although the treatments are becoming diversified, the results are still not satisfactory. Thus, it is necessary to develop alternative effective target to improve treatment efficiency and reduce side effects for CRC.

In meiosis and mitosis, the separation of chromosomes and the normal division of cells are inseparable from the centromere [9]. Previously, more than 40 centromere proteins have been identified [10]. Homo sapiens centromeric protein O (CENPO, NCBI Reference Sequence: NM_024322.3), also known as ICEN36, located on the centromere [11]. CENP-O, -P, -Q, -R and -50 are all defined as CENPO proteins [12]. CENPO prevents premature separation of sister chromatids during spindle injury recovery, which is associated with cell death [12]. Small interference (Si) RNA transfer of CENPO protein will lead to the increase of aneuploidy and aneuploidy chromosomes, which will lead to disease or cancer [10]. Recent studies have shown that the alteration of CENPO expression can mediate the proliferation of gastric cancer cells [13]. However, the direct role of CENPO in cancer and the detailed molecular mechanism are currently unclear, especially in CRC. Therefore, the object of this fundamental research will be revealing the role of CENPO in CRC.

This study identified the difference in CENPO expression between cancer and normal tissues in patients with CRC. Subsequently, proliferation, cycle distribution, apoptotic, migration and invasion of CRC cells were detected in loss-of-function assays in vitro. The effects of CENPO knockdown on CRC was evaluated in mice xenograft model. Moreover, the potential mechanism of CENPO in CRC was preliminarily explored.

## Materials and methods

### Immunohistochemical (IHC) staining

The approval of all experimental procedures related to human CRC samples comes from the ethics committee of Sun Yat-sen Memorial Hospital of Sun Yat-sen University and follows relevant guidelines and regulations. The 100 pairs of cancer tissues and matched non-cancer tissues of CRC patients (Shanghai Outer Biotechnology Company) were used to characterize the expression level of CENPO through IHC experiments. These tissues were fixed with 4% formalin, made into paraffin-embedded sections, dewaxed with xylene, hydrated with alcohol, repaired with sodium citrate, and soaked into 3% H_2_O_2_ to remove endogenous catalase. Subsequently, the tissue was eluted with PBS and incubated with the primary antibody against CENPO (1:200, Biorbyt, USA, #orb335144) at 4 °C for 3 h and secondary antibody (1: 400, Abcam, USA, #ab6721) at room temperature for 2 h in sequence. After that, the tissues were treated with DAB, stained with hematoxylin, dehydrated with ethanol gradient, dewaxed with xylene, dried, sealed with neutral gum, and observed under microscope (Nikon C2  +  Confocal Microscope, Japan) with magnification of 200 and 400. Notably, high or low expression of CENPO was defined by the median of the scores of each tissue IHC experiment.

### Cell culture condition

The human CRC cell line HCT116 and RKO (Cell Bank of the Chinese Academy of Sciences, Shanghai, China) were placed in an incubator (SANYO) at 37 °C with moist air containing 5% CO_2_, supplemented with Dulbecco’s Modified Eagle’s Medium (DMEM, GIBCO) and 10% fetal bovine serum (FBS, GIBCO).

### Establishment of CRC cell lines with CENPO knockdown

CENPO, the mRNA of transcriptional variant 1 was used to design three RNA interference target sequences (Table S1). The fragment was digested by AGE I (5′-ACCGGT-3′, 10 U/µL, NEB) and EcoR I (5′-GAATTC-3′, 10 U/µL, NEB) and inserted into linearized vector BRV-112 (5′-CCGGTTCTCCGAACGTGTCACGTTTCAAGAGAACGTGACACGTTCGGAGAATTTTTG-3′) (BIOSCIRES, Shanghai, China) using T4 DNA Ligase (Fermentas). Notably, the green fluorescent protein (GFP) tag of the lentiviral vector BRV-112 was used to estimate the transfection efficiency. Subsequently, 5 × 10^6^ HCT116 and RKO cells were co-transfected with 10 μg recombinant BRV-112 plasmids at a multiplicity of infection of 10 using Lipofectamine 3000 (Invitrogen) for 1 h. After transfection with lentivirus, cell transfection efficiency was evaluated under fluorescence microscope (OLYMPUS).

### Quantitative-PCR (qPCR) analysis

According to the kit instructions (Invitrogen, Carlsbad, CA, USA), RNA extraction from lentivirus-transfected HCT116 and RKO cells. RNA was reverse transcribed into cDNA using the Promega M-MLV kit. Subsequently, template cDNA was used for qPCR analysis with Ace Q qPCR SYBR Green Master Mix (Vazyme, Nanjing, China). The relative mRNA expression of CENPO was quantified with cycle threshold (Ct) values and normalized using the 2^−∆∆Cq^ method. Notably, the primer sequence, and the size of the amplicon synthesized after qPCR were summarized in Table S2. GAPDH as a reference control.

### Western blot

Total protein from lentivirus-transfected HCT116 and RKO cells was extracted using RIPA (Beyotime) and the protein concentration was detected by BCA Protein Assay Kit (Beyotime). Western analysis was separated using SDS-PAGE (10%), transferred to polyvinylidene difluoride (PVDF) membrane, blocked with 5% BSA and 0.5% Tween 20 at 4 °C for 1 h. Next, the protein was co-incubated with the primary antibodies (Table S3) at 4 °C for 3 h and then with the goat anti-rabbit IgG polyclonal antibody (1:3000) labeled with horseradish peroxidase (HRP) at room temperature for 2 h. Finally, protein signal was visualized using chemiluminescence ECL-PLUS kit (Thermo Fisher Scientific).

### MTT assay

After digesting the lentivirus-transfected (shCtrl, shCENPO) HCT116 and RKO cells with trypsin, the cells were resuspended into cell suspension and counted with a counting plate (Cellometer, Cat. #SD-100). The cells were cultured into 6-well plates at a density of 2000 cells/well for 5 days, with 3 replicates in each group. On the second day after plate laying, 20 μL 5 mg/mL MTT and 100 μL DMSO were added in turn for 2–5 min and then placed in the enzyme plate analyzer to detect OD490 nm value.

### Celigo cell counting assay

After digesting the HCT116 and RKO cells [shCtrl, shCENPO, shCENPO  +  AKT activation (10 μM, SC79, MEC, Cat. #HY-18749)] with trypsin, the cells were resuspended into cell suspension and counted with a counting plate (Cellometer, Cat. #SD-100). The cells were cultured into 6-well plates at a density of 2000 cells/well and the culture system was 100 μL/ well. Celigo (Nexcelom) was monitored once a day for 5 consecutive days. The number of cells was accurately calculated according to the amount of GFP in each scanning orifice. The 5-day data were calculated and the cell proliferation curve was plotted.

### Flow cytometry cell cycle assay

Lentivirus-transfected (shCtrl, shCENPO) HCT116 and RKO cells were inoculated in 6-well plates (2 mL/well) for 5 days. The cells were eluted by PBS, centrifuged for 5 min at 200*g*, fixed with ethanol, and stained with propidium iodide (PI). The ratio of cells in the G1, S and G2 phases of the CENPO knockdown group and the control group were detected and analyzed by flow cytometry.

### Flow cytometry apoptotic assay

Lentivirus-transfected (shCtrl, shCENPO) HCT116 and RKO cells were inoculated in 6-well plates (2 mL/well) for 5 days. After centrifugation, the cells were successively washed and precipitated by PBS and 1 ×  binding buffer, stained with 5 μL annexin V-APC in the dark for 15 min. The apoptosis rate was detected by flow cytometry and the results were analyzed by T test.

### Human apoptosis antibody array

Follow the apoptotic antibody array kit (Abcam, USA, #ab134001) instructions, lentivirus-transfected (shCtrl, shCENPO) HCT116 cells protein was diluted with the array diluent buffer kit to 0.5 mg/mL. Subsequently, the array antibody membrane was blocked using blocking buffer at room temperature for 30 min, incubated with HRP linked streptavidin at 4 °C overnight. ChemiDoc XRS chemiluminescence detection and imaging system were used to visually detect proteins.

### Wound-healing assay

Lentivirus-transfected (shCtrl, shCENPO) HCT116 and RKO cells were inoculated in 6-well plates (100 μL/well) at a density of 5 × 10^4^ cells/well for 5 days. In brief, vertical lines for each well were drawn at 0 h, 24 h and 48 h using a pipette. After incubation, the cells were washed with PBS, fixed with 3.7% paraformaldehyde (Corning) for 15 min, and stained with 1% crystal violet (Corning) for 10 min. Finally, cells were placed under a microscope for image acquisition and Image J software (National Institutes of Health) was used to quantify the distance (μm) between the scratches at different time points.

### Transwell invasion assay

Lentivirus-transfected (shCtrl, shCENPO) HCT116 and RKO cells were placed into Transwell chambers (24-well, 8-mm pore) (Corning) at a density of 80,000 cells/well for incubation24 h at 37 °C. The inner compartment contained 100 μL cell suspension and the outer compartment was 500 μL DMEM medium containing 30% FBS. After that, non-invading cells on the upper chamber were removed, the cells attached to the polycarbonate membrane were fixed with 4% precooled paraformaldehyde for 30 min and stained with 0.1% crystal violet at room temperature for 20 min. Afterwards, the cells were placed under a 200 ×  microscope to capture images from five randomly selected fields.

### Establishment of animal xenograft model

The approval of animal experimental procedures came from the ethics committee of Sun Yat-sen University and followed relevant guidelines and protocols for animal care and protection. Lentivirus-transfected (shCtrl, shCENPO) RKO cells 500 μL were digested with trypsin, resuspended and injected into the right forearm of BALB/c female nude mice (8 × 10^6^ cells/mouse) (Jiesijie Experimental Animals Co., Ltd., Shanghai, China). With 10 mice per group, tumorigenesis rates in the shCtrl group and the shCENPO group were 50% and 20%, respectively. Mice were anesthetized with 0.7% sodium pentobarbital (10 μL/g) and placed in the IVIS spectral fluorescence imaging system (emission wavelength of 510 nm) to assess tumor burden. Tumor size was estimated every other week until 20 days after subcutaneous injection. 26 days later, the mice were treated with cervical dislocation, tumors were taken out, weighed, and photographed. Finally, the expression of CENPO (1:200, Biorbyt, USA, #orb335144), Ki67 (1: 200, Abcam, USA, #ab16667), AKT (1:200, Proteintech, USA, #60203-2-Ig) and p-AKT (1:200, MILLIPORE, USA, #05-1003) was detected by IHC staining in mouse tumor tissues of shCENPO group and shCtrl group as previous described. Images were observed under microscope (Nikon C2  +  Confocal Microscope, Japan) with magnification of 200.

### Statistical analysis

The results presented represent experiments repeated at least 3 times and are expressed as mean  ±  SD. Statistical was analyzed using GraphPad Prism 7.0 software (GraphPad Software Inc., San Diego, CA, USA) and SPSS 21.0 (IBM, Armonk, NY, USA). All tests were analyzed using paired t test and one-way ANOVA followed by Bonferroni’s post hoc test analysis. *P*  < 0.05 were considered statistically significant.

## Results

### CENPO is abundantly expressed in CRC

**Fig. 1:**
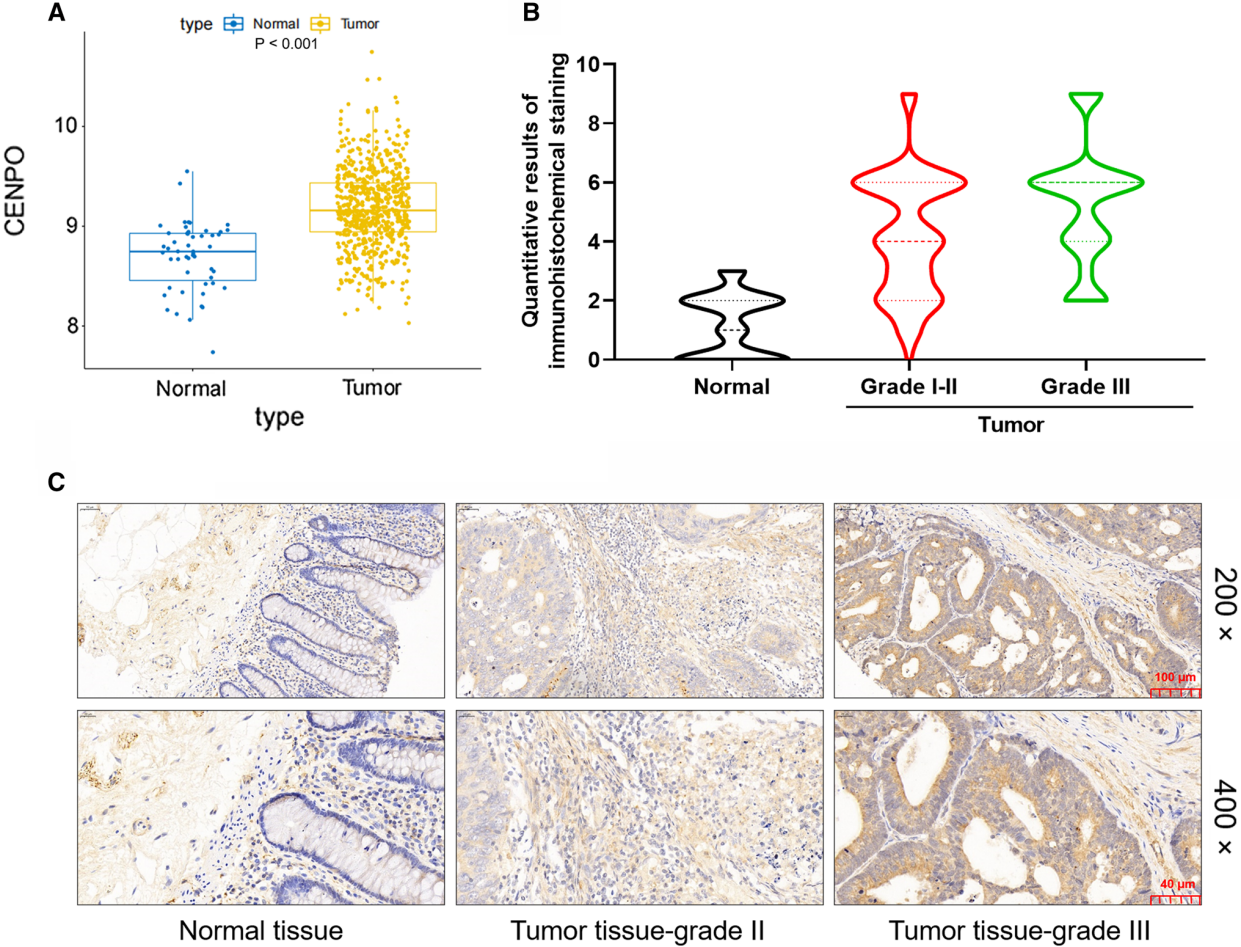
CENPO was highly expressed in CRC. **A** Based on the information of 51 normal and 635 tumor samples from CRC patients in The Cancer Genome Atlas (TCGA) database, the differential expression of CENPO was analyzed. **B** The quantitative analysis results of CENPO in clinical tissues of IHC. **C** The typical images of IHC staining showed a significantly high signal intensity of CENPO in tumor tissue. Magnification was 200 and 400

Based on the information of 51 normal and 635 tumor samples from CRC patients in The Cancer Genome Atlas (TCGA) database, the differential expression of CENPO was analyzed. As shown in Fig. 1A, the expression level of CENPO in tumors is significantly higher than that in normal tissues (Fig. 1A). In addition, the Kaplan–Meier analysis of TCGA-based sample information showed that the survival time of patients with high expression of CENPO was reduced. In view of the limited sample size, the expression level of CENPO had no significant effect on the survival time of CRC patients (Fig. S1A). Subsequently, we performed IHC staining in the normal and tumor tissues of clinical CRC patients to further clarify the expression of CENPO in CRC. According to the results of the IHC score, the value greater than the median of 3 was defined as the high expression of CENPO, otherwise it was the low expression (Fig. 1B). We found that the proportion of high expression of CENPO in tumor tissues of CRC patients was significantly higher than that of normal tissues (*P*  < 0.001) (Table 1). Consistently, the typical images of IHC staining showed a higher signal intensity of CENPO in tumor tissue than the normal tissue (Fig. 1C). Moreover, the relationship between CENPO expression level and tumor characteristics of CRC patients was characterized by Mann–Whitney U (Table 2). The results suggested that CENPO expression was significantly positively correlated with tumor grade. Pearson correlation further indicated that the increased expression of CENPO predicted the deepening of tumor malignancy (Table 3). Taken together, CENPO expression was not only highly expressed in tumor tissues, but also positively correlated with the deterioration of CRC patients.

**Table 1:** Expression patterns in colorectal cancer tissues and normal tissues revealed in immunohistochemistry analysis

CENPO expression	Tumor tissue		Normal tissue		*P* value
	Cases	Percentage (%)	Cases	Percentage (%)	
Low	46	51.1	78	100	0.000***
High	44	48.9	0	–	

*P < 0.05, ***P < 0.001

**Table 2:** Relationship between CENPO expression and tumor characteristics in patients with colorectal cancer

Features	No. of patients	CENPO expression		*P* value
		Low	High	
All patients	90	46	44	
Age (years)				0.917
≤ 67	45	23	22	
> 67	44	22	22	
Gender				0.344
Male	53	29	24	
Female	36	16	20	
Grade				0.033*
II	70	40	30	
III	20	6	14	
T infiltrate				0.215
T2	2	0	2	
T3	60	30	30	
T4	28	16	12	
Lymphatic metastasis (N)				0.628
N0	45	24	21	
N1	36	18	18	
N2	9	4	5	
AJCC stage				0.835
1	2	0	2	
2	42	23	19	
3	42	20	22	
4	4	3	1	
Tumor size				0.327
≤ 5 cm	52	24	28	
> 5 cm	37	21	16	
Lymph nodes				1.000
< 7	45	23	22	
≥ 7	45	23	22	
Lymph node positive				0.675
< 1	45	24	21	
≥ 1	45	22	23	

*P < 0.05, ***P < 0.001

**Table 3:** Relationship between CENPO expression and tumor characteristics in patients with colorectal cancer

CENPO	*P* value
Grade	
Pearson correlation	0.226
Significance (double-tailed)	0.032*
N	90

*P < 0.05, ***P < 0.001

### CENPO is downregulated in shRNA-mediated CRC cell lines HCT116 and RKO

**Fig. 2:**
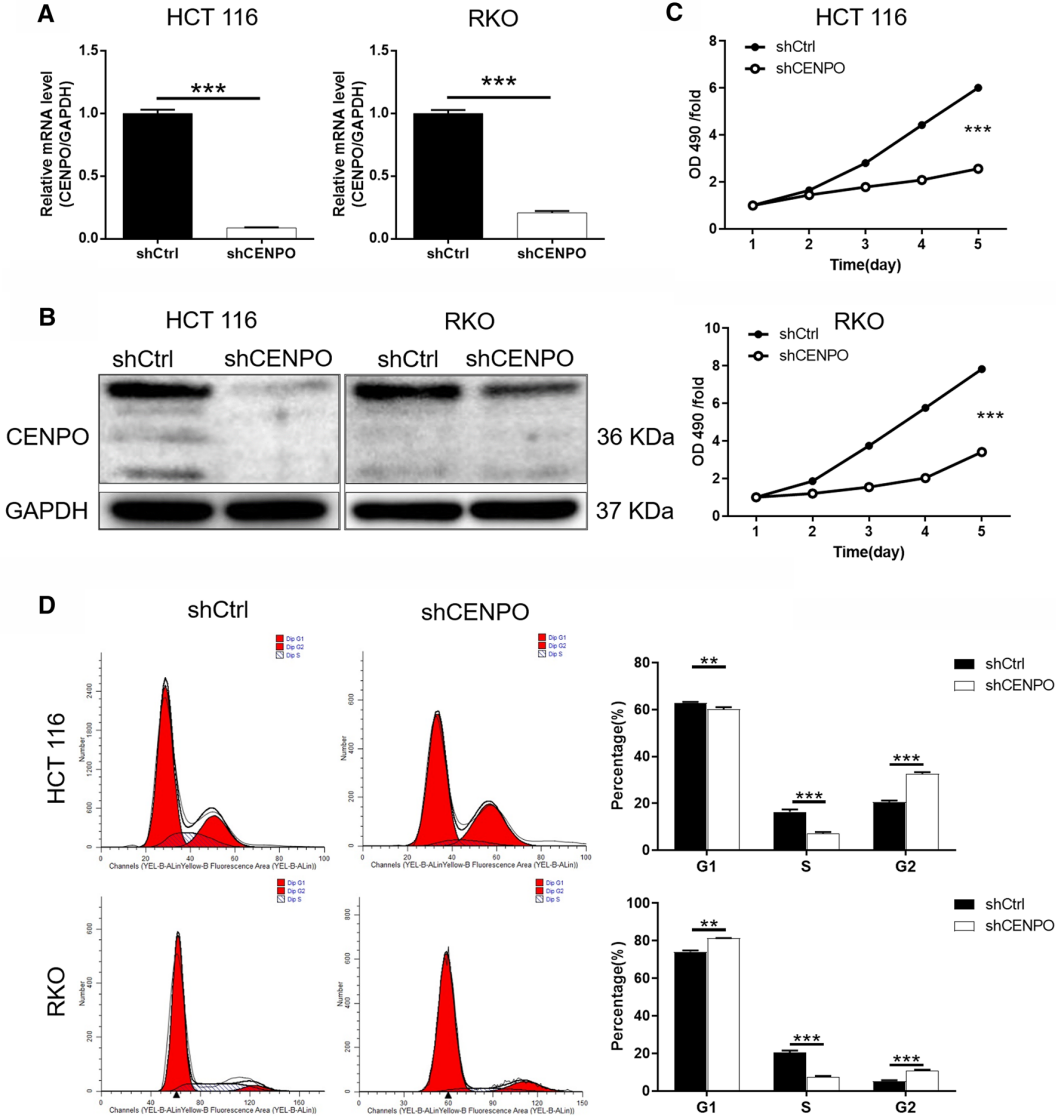
Knockdown of CENPO inhibited CRC cells proliferation and arrested cell cycle in G2. The mRNA (**A**) and protein (**B**) expression levels of CENPO in HCT116 and RKO cells after lentiviral (shCtrl and shCENPO) interference were estimated respectively. **C** The effect of CENPO knockdown on HCT116 and RKO cell proliferation was detected using MTT assay. **D** Flow cytometry with PI staining was used to detect the effect of CENPO knockdown on the cell cycle distribution of HCT116 and RKO cells. The presented results were representative of experiments repeated at least three times. Data was represented as mean  ±  SD. *P  < 0.05, **P  < 0.01, ***P  < 0.001

To further characterize the effect of CENPO on CRC, shRNA-mediated HCT116 and RKO cells were established. As illustrated in Fig. S1B, the knockdown efficiency of CENPO in shCENPO-1 group was the highest (80.5%) compared with the other groups (*P*  < 0.01). Subsequently, shCENPO-1 was used for CENPO knockdown in HCT116 and RKO cells. After the cells were transfected with shCtrl or shCENPO for 72 h, the high expression of GFP in HCT116 and RKO cells indicated successful transfection of lentivirus shCENPO (Fig. S1C). Subsequently, the knockdown efficiency of CENPO in shCENPO group were 91.3% and 79.3% in HCT116 and RKO cell, respectively (*P*  < 0.001) (Fig. 2A). Moreover, western blot displayed that proteins level of CENPO showed a sharp decrease in shCENPO group compared with shCtrl group (Fig. 2B and Fig. S2A). These results indicated that the cell models of CENPO knockdown were successfully constructed.

### Downregulation of CENPO inhibits CRC cell proliferation and arrests cell cycle in G2

HCT116 cells and RKO cells transfected with shCENPO and shCtrl, respectively, and estimated for cell viability using MTT assay. The results showed that the OD 490/fold of HCT116 and RKO cells in shCENPO group were significantly lower than that in shCtrl group (P  < 0.001) (Fig. 2C), suggesting that decreased expression of CENPO attenuated cell viability. Meanwhile, the cell cycle of HCT116 and RKO was analyzed by flow cytometry after downregulation of CENPO. Compared with the control group, the proportion of S phase cells decreased, while the proportion of G2 phase HCT116 cells increased (*P*  < 0.001). Not surprisingly, the same phenomenon was also observed in RKO cells (*P*  < 0.01) (Fig. 2D). Collectively, CENPO-knocked down CRC cells could inhibit cell proliferation and arrest cycle in G2 phase.

### Downregulation of CENPO induces apoptosis of CRC cells

**Fig. 3:**
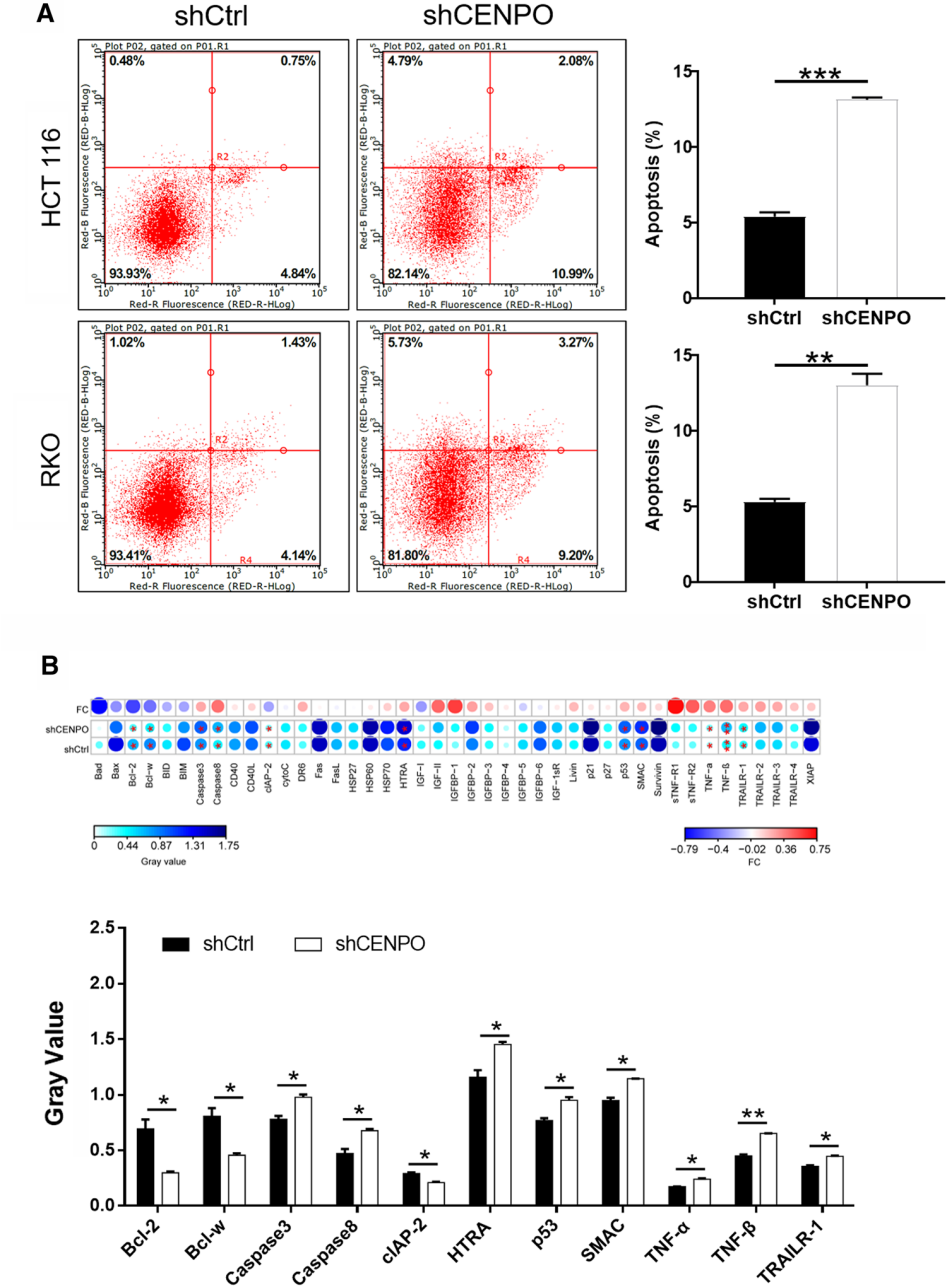
Knockdown of CENPO enhanced apoptosis in CRC cells. **A** Flow cytometry analysis based on Annexin V-APC and PI staining was utilized to detect the apoptotic for HCT116 and RKO cells. **B** The alterations of apoptotic signaling pathway were preliminarily explored in CENPO knock downed HCT116 cells through Human apoptosis antibody array analysis. The presented results were representative of experiments repeated at least three times. Data was represented as mean  ±  SD. *P  < 0.05, **P  < 0.01, ***P  < 0.001

Apoptosis alteration is responsible not only for tumor development and progression but also for tumor resistance to therapies. In this study, flow cytometry and TUNEL assay were used to investigate the effect of CENPO expression on CRC cell apoptosis [14]. The results of flow cytometry showed that apoptosis rate was three-fold higher in shCENPO than that in shCtrl group of HCT116 cells (*P*  < 0.001). Meanwhile, the apoptosis rate in shCENPO group was twice as high as that in shCtrl group in RKO cells (*P*  < 0.001) (Fig. 3A). Furthermore, the expression of apoptosis related proteins was detected after CENPO expression was reduced. Among the 43 detected proteins, pro-apoptosis proteins including Caspase3, Caspase8, HTRA, p53, SMAC, TNF-α, TNF-β and TRAILR-1 were significantly upregulated, while anti-apoptosis proteins Bcl-2, Bcl-w and CLAP-2 were significantly downregulated (P  < 0.05) (Fig. 3B). As a consequence, the decrease of CENPO expression not only promoted apoptosis but also resulted in the alteration of apoptotic related protein.

### Downregulation of CENPO inhibits the migration and invasion of CRC cells through EMT

**Fig. 4:**
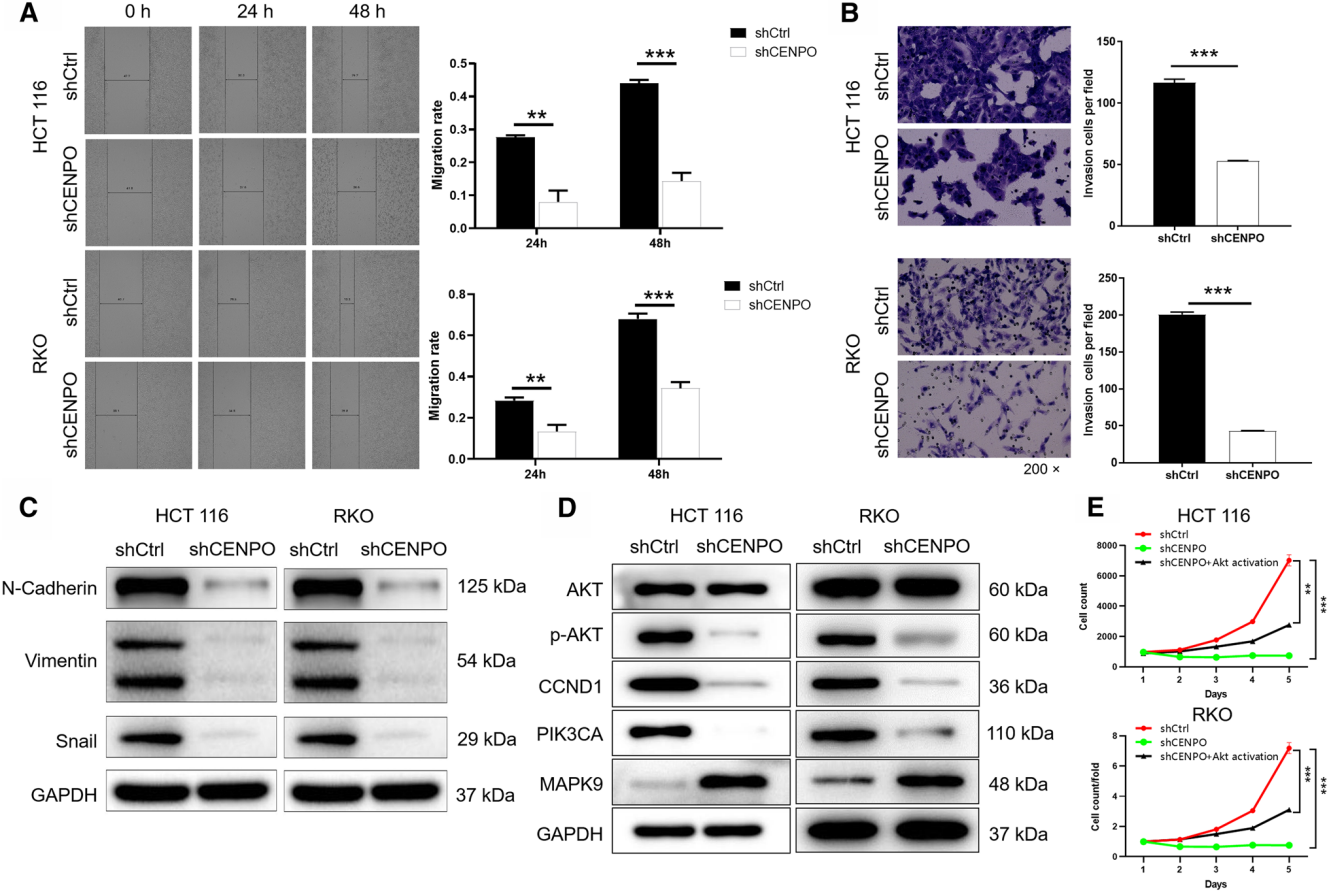
Knockdown of CENPO migration and invasion of CRC cells. The effect of CENPO knockdown on HCT116 and RKO cell migration (**A**) and invasion (**B**) was evaluated using wound-healing assay (**A**) and Transwell assay (**B**). **C** The expression of EMT-related protein of HCT116 and RKO cells with or without knockdown of CENPO by Western blot. **D** The expression of target proteins pathways was observed by Western blot in HCT116 and RKO cells. **E** The RKO cells with AKT activator on the basis of CENPO knockdown (shCENPO  +  AKT activator), which was used to detect the proliferation ability of cells. The presented results were representative of experiments repeated at least three times. Data was represented as mean  ±  SD. *P  < 0.05, **P  < 0.01, ***P  < 0.001

As showed in Fig. 4A, the migration ability of HCT116 and RKO cells in the shCENPO group within 48 h decreased by 66% and 49%, respectively, compared with the shCtrl group (*P*  < 0.001). Consistently, Transwell assays showed that numbers of invasion HCT116 and RKO cells were significantly reduced after knockdown of CENPO (*P*  < 0.001) (Fig. 4B). Based on these results, we inferred that knockdown of CENPO can suppress the migration and invasion of CRC cell lines HCT116 and RKO. In addition, the invasion and metastasis of tumor cells usually requires the process of epithelial-mesenchymal transition (EMT) [15]. N-cadherin, Vimentin, and Snail are regarded as the most common markers in the EMT process [16]. The present study demonstrated that knock down of CENPO downregulated the expression of N-cadherin Vimentin, and Snail (Fig. 4C and Fig. S2B). Thus, downregulation of CENPO inhibited the migration and invasion of CRC cells through EMT.

### Downregulation of CENPO inhibits progression of CRC via PI3K/AKT signaling pathway

In addition, we briefly summarized the results of exploration on the downstream signaling pathway. The reduced expression of CENPO attenuated the phosphorylation level of AKT, downregulated CCND1, PIK3CA, and upregulated MAPK9 (Fig. 4D and Fig. S2C). In addition, the correlation between CENPO and AKT, CCND1, PIK3CA and MAPK9 was initially analyzed through Pearson’s correlation. The results indicated that CENPO was positively correlated with the expression levels of AKT1, AKT2, AKT3, CCND1, PIK3CA, and MAPK9 (Fig. S2D). However, the correlation between CENPO and the tumor proteins mentioned in the text required further experimental verification. Furthermore, the RKO cells with AKT activator on the basis of CENPO knockdown (shCENPO  +  AKT) was used to detect the proliferation ability. As shown in the Fig. 4E, the cell proliferation ability of shCENPO group was inhibited most strongly (fold change  = 9.5, *P*  < 0.001), shCENPO  +  AKT activator group could slow down the inhibitory effect of shCENPO group (fold change  = 2.3, *P * < 0.001) (Fig. 4E). Therefore, we suggested that CENPO knockdown inhibits CRC by mediating PI3K/AKT signaling pathway.

### *Downregulation of CENPO impaired tumor formation *in vivo

**Fig. 5:**
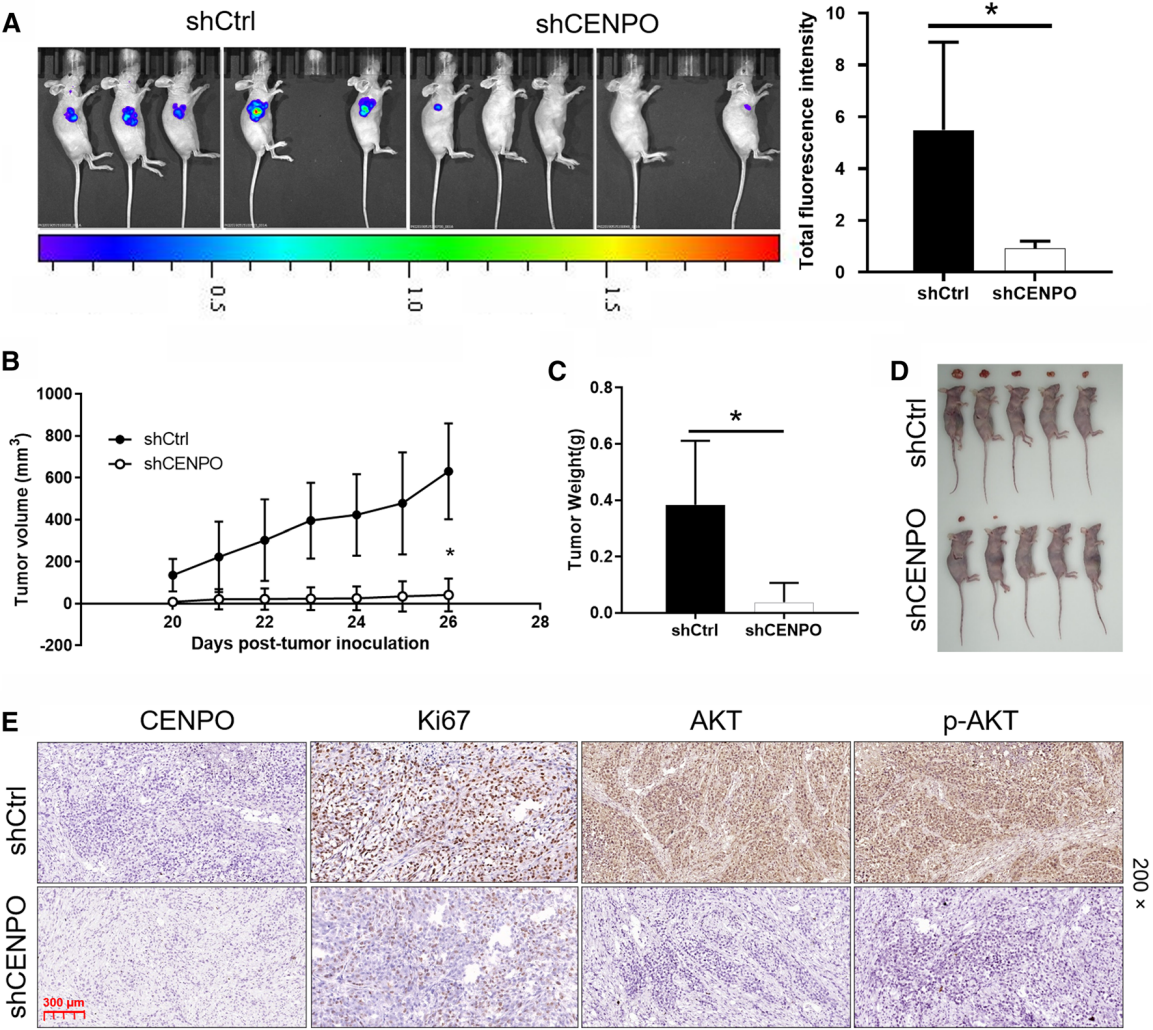
Knockdown of CENPO inhibited tumor growth in mice xenograft models. The mice xenograft model was established to observe the effects of CENPO knockdown on fluorescence expression intensity (**A**), tumor volume (**B**) and weight (**C**, **D**). **E** The expression of CENPO, Ki67, AKT and p-AKT was detected by IHC staining in mouse tumor tissues of shCENPO group and shCtrl group. The presented results were representative of experiments repeated at least three times. Data was represented as mean  ±  SD. *P  < 0.05, **P  < 0.01, ***P  <  0.001

The mice xenograft model was established to further determine the effect of CENPO in CRC in vivo. The monitoring results on the 26th day showed that the fluorescence intensity of tumor in shCENPO group was significantly higher than that in shCtrl group (*P*  < 0.05) (Fig. 5A). Moreover, the tumor volume in the shCENPO group has been growing, while the growth in the shCtrl group has almost stagnated (*P*  < 0.05) (Fig. 5B). Not surprisingly, the average tumor weight in the shCENPO group was 346.00 ± 158.00 mg, which was lower than that in the shCtrl group (*P*  < 0.05) (Fig. 5C, D). Furthermore, the expression of CENPO, Ki67, AKT and p-AKT was detected by IHC staining in mouse tumor tissues of shCENPO group and shCtrl group. As shown in the Fig. 5E, the signal intensity of CENPO, Ki67, AKT and p-AKT in the tumor tissue after CENPO knockdown was reduced. As a consequence, these results suggested that the downregulation of CENPO expression can lead to the weakened growth of mouse xenograft tumors.

### The function of CENPO in regulating proliferation and apoptosis depended on p53

**Fig. 6:**
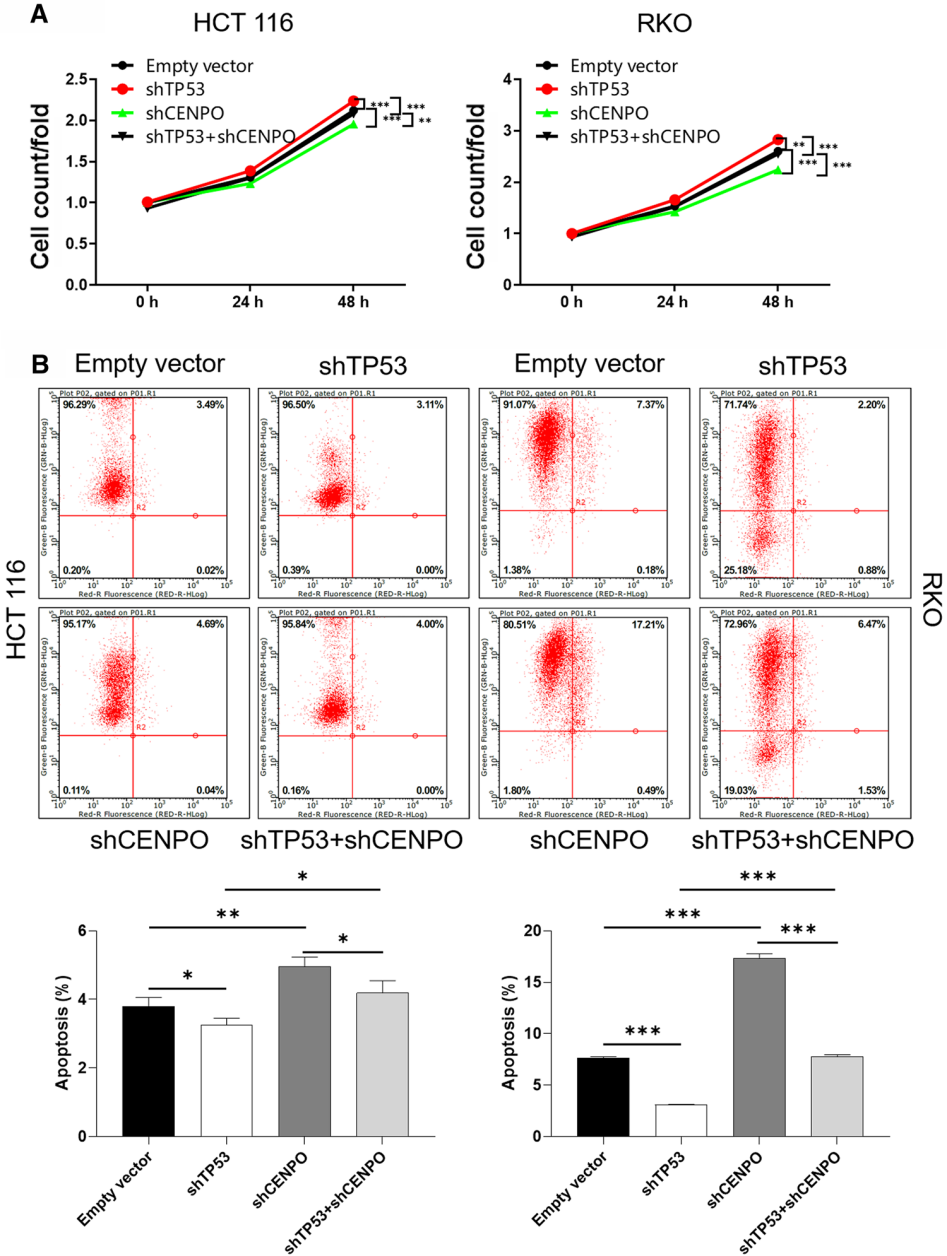
CENPO regulated proliferation and apoptosis in a p53-dependent manner. **A**, **B** Cell counting experiment and flow cytometry were performed to analyze cell proliferation and apoptosis of HCT116 and RKO cells co-transfected with TP53 shRNA and TC2N shRNA (shTP53  +  shCENPO). The presented results were representative of experiments repeated at least three times. Data was represented as mean  ±  SD. *P  < 0.05, **P  < 0.01, ***P  < 0.001

We next examined whether p53 mediated the effect of CENPO on tumorigenesis in CRC cells. The expression of p53 was blocked using shRNA (shTP53) in HCT116 and RKO cells with the simultaneous knockdown of CENPO (shTP53  +  shCENPO). Subsequently, cell proliferation and apoptosis were assessed. We found that the knockdown of p53 by shRNA significantly abrogated the effects on cell proliferation and apoptosis induced by CENPO knockdown in HCT116 and RKO cells (Fig. 6A, B). Thus, our data suggested that CENPO regulated proliferation and apoptosis in a p53-dependent manner.

## Discussion

Centromere is an important component of chromosome separation during meiosis and mitosis of normal cells [9]. Previous study has reported that abnormal localization or overexpression of the centromere protein CENPO leads to cell division disorders and chromosomal aneuploidy [11]. The biological process is closely related to the progression of cancers [17]. For instance, Cao et al. reported that the expression of CENPO was not only related to the prognosis of gastric cancer patients, but also regulated the proliferation of gastric cancer cells [13]. Moreover, CENPO regulated the cell cycle by mediating mitotic spindle assembly and participated in the BC process [18]. The present study demonstrated the role of CENPO in promoting CRC. IHC staining used to identify the difference in CENPO expression between cancer and normal tissues in patients with CRC. We found that expression of CENPO was highly expressed in tumor tissues of CRC patients. Expression of CENPO was positively correlated with the deterioration of CRC patients. Additionally, downregulation of CENPO inhibited the malignant progression of CRC cells, such as reduced proliferation, cycle repression in G2 phase, enhanced apoptotic sensitivity and inhibition of migration.

Previous study demonstrated that the migration and metastasis of tumor cells usually required the process of epithelial-mesenchymal transition (EMT) [15]. N-cadherin, E-cadherin, and Vimentin are regarded as the most common markers in the EMT process [16]. Choi et al. suggested that the metastasis of CRC cells required the involvement of EMT, such as the abnormal expression of N-cadherin and Vimentin [19, 20]. Similarly, MicroRNA-1275 regulated Vimentin and E-cadherin to inhibit the migration and invasion of gastric cancer cells [21]. The study showed that knockdown of CENPO contributed to downregulation of N-cadherin and Vimentin, upregulation of E-cadherin. Thus, CENPO mediated the process of EMT to regulate the migration and invasion of CRC cells.

This study indicated that decrease of CENPO expression can induce apoptosis of CRC cells. It has been pointed out that the reduction of cell apoptosis is an important factor of tumor genesis and carcinogenesis [22]. The initiation of apoptosis is an extremely complex process, which is inseparable from the interaction of internal and external pathways [23, 24]. Generally speaking, the intrinsic pathway is activated by various stimuli, such as DNA damage and loss of cell survival factors, which are controlled by a series of Bcl family members. The binding of the death ligand to the receptor triggers an external pathway [25]. The present study indicated that knockdown of CENPO contributed to upregulation of Caspase3, Caspase8, HTRA, p53, SMAC, TNF-α, TNF-β and TRAILR-1. On the contrary, Bcl-2, Bcl-w and CLAP-2 were downregulated. The activation of Caspase8 can promote the rupture of Caspase3 and PARP, and activate mitochondrial-mediated endogenous apoptosis [26]. Cong et al. identified that upregulated expression of Caspase3 induced apoptosis of CRC cells [26]. In addition, HTRA induces apoptosis by degrading XIAP and activating PI3K/AKT pathway [26]. Activation of p53 can induce tumor apoptosis and enhance the response of CRC cells to chemotherapy drugs 5-Fluorouracil and Oxaliplatin [27]. Hehlgans and Grimm et al. pointed that either SMAC or TRAILR-1 can induce apoptosis of CRC cells inhibit DNA damage repair [28, 29]. Furthermore, Shen et al. and Buhrmann et al. clarified that TNF-*α*, TNF-*β* are similar [30], they can induce tumor-related apoptosis to inhibit metastasis of CRC cells [31, 32]. Huang et al. and Gibson et al. suggested that high expression of anti-apoptotic protein Bcl-2 and Bcl-w are prognostic factors for CRC [33–35]. CLAP-2, also known as CLASP, maintains cell proliferation by regulating the dynamic stability of microtubules [36, 37]. In this study, we found that knockdown of CENPO contributed to upregulation of pro-apoptosis proteins and downregulation of anti-apoptosis proteins. The alterations of apoptotic signaling pathway were preliminarily explored through the occurrence of apoptosis in tumor cells. Of course, the process of apoptosis signaling pathway required to be further explored, which will provide an important theoretical basis for cancer treatment strategies.

The ability of knockdown CENPO to inhibit tumorigenesis has prompted us to explore downstream pathways that may mediate the carcinogenesis of CENPO. In this study, we demonstrated that CENPO knockdown decreased expression of downstream protein p-AKT, CCND1, PIK3CA, while MAPK9 was increased. Previous study had proved that AKT kinase activation (p-AKT) plays a central role in regulating transcription, cell survival and apoptosis, and is one of the prognostic factors of CRC [38, 39]. CCND1 regulates DNA repair, and overexpression of this protein may be related to the poor clinical prognosis and distant metastasis of CRC patients, which are considered as the poor prognosis indicator of CRC [40, 41]. PIK3CA (PI3K) mutation is also a common feature of CRC, which is related to poor prognosis [42, 43]. Both MAPK and PI3K pathways are involved in CRC cells proliferation and survival. Yaeger et al. illuminated that inhibition of MAPK/PI3K pathway is more effective in the treatment of metastatic CRC [44]. Consequently, CENPO is implicated in CRC cell progression by regulating downstream pathways p-AKT, CCND1, PIK3CA and MAPK9.

This study demonstrated for the first time the promoting effect of CENPO in CRC. CENPO was not only highly expressed in tumor tissues, but also positively correlated with the deterioration of CRC patients. Additionally, downregulation of CENPO inhibited the malignant progression of CRC cells, such as reduced proliferation, cycle repression in G2 phase, enhanced apoptotic sensitivity and inhibition of migration. CENPO mediated the process of EMT to regulate the migration and invasion of CRC cells. The reduced expression of CENPO attenuated the phosphorylation level of AKT, downregulated CCND1, PIK3CA, and upregulated MAPK9. In vivo experiments further confirmed that CENPO downregulation attenuated tumor growth. In summary, the prominent discovery was the determination of the promoting role of the CENPO in CRC, demonstrating that small molecule inhibitors targeting CENPO were a novel therapeutic strategy for CRC.

## Supplementary Information

Below is the link to the electronic supplementary material.

Supplementary file 1 (DOCX 1490 kb)

## Data Availability

The data used and/or analyzed during the current study are available from the corresponding author on reasonable request.
