# Supplementary material for: CENPO regulated proliferation and apoptosis of colorectal cancer in a p53-dependent manner
Source: Discov Oncol. 2022 Feb 3;13:8. doi: 10.1007/s12672-022-00469-2 (PMC8810981; doi:10.1007/s12672-022-00469-2)
Supplement: Supplementary file 1 — (DOCX 1490 kb) [file 12672_2022_469_MOESM1_ESM.docx]

**
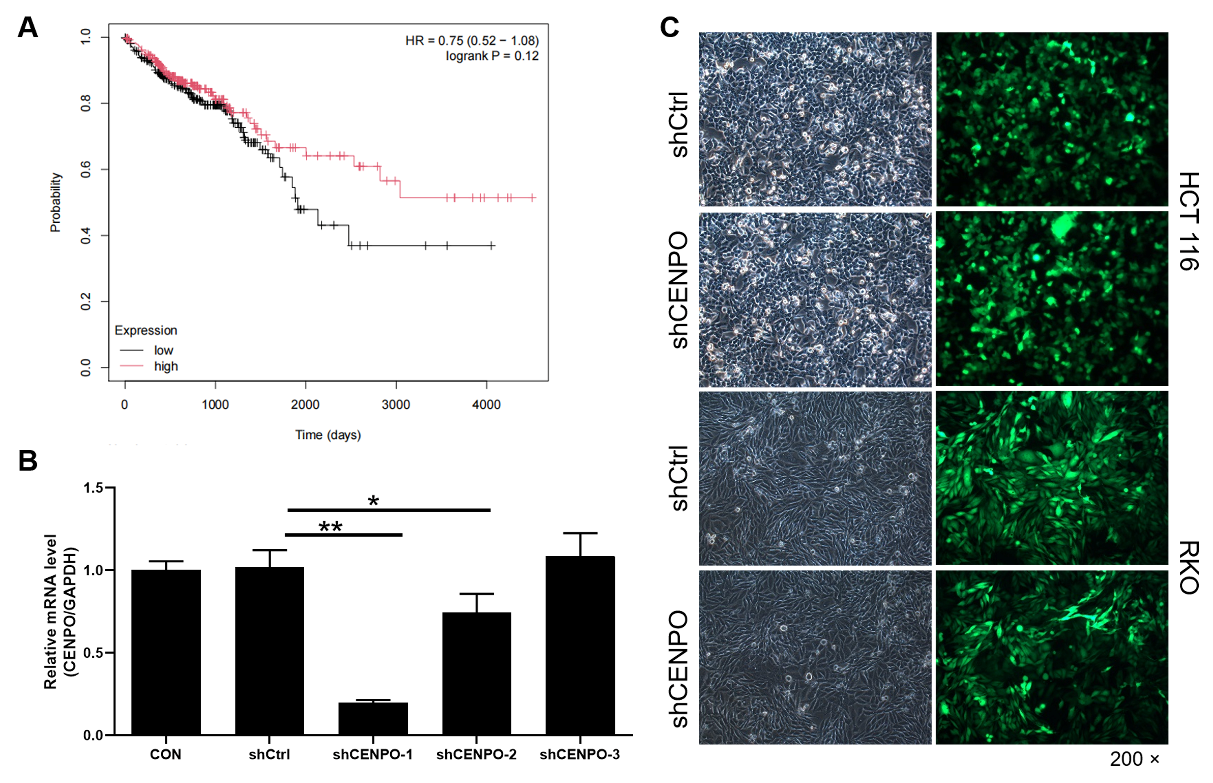
**

**Fig S1. Knockdown of CENPO in CRC cells.**

(A) The correlation between CENPO expression level and survival of CRC patients was analyzed by Kaplan-Meier method based on TCGA sample information. (B) The effective sequence of shRNA lentivirus-mediated interference with CENPO expression was screened. (C) The expression of GFP after transfection of lentiviral shRNA and shCENPO into HCT116 and RKO was observed under a fluorescence microscope. The presented results were representative of experiments repeated at least three times. Data was represented as mean ± SD. *P < 0.05, **P < 0.01, ***P < 0.001.


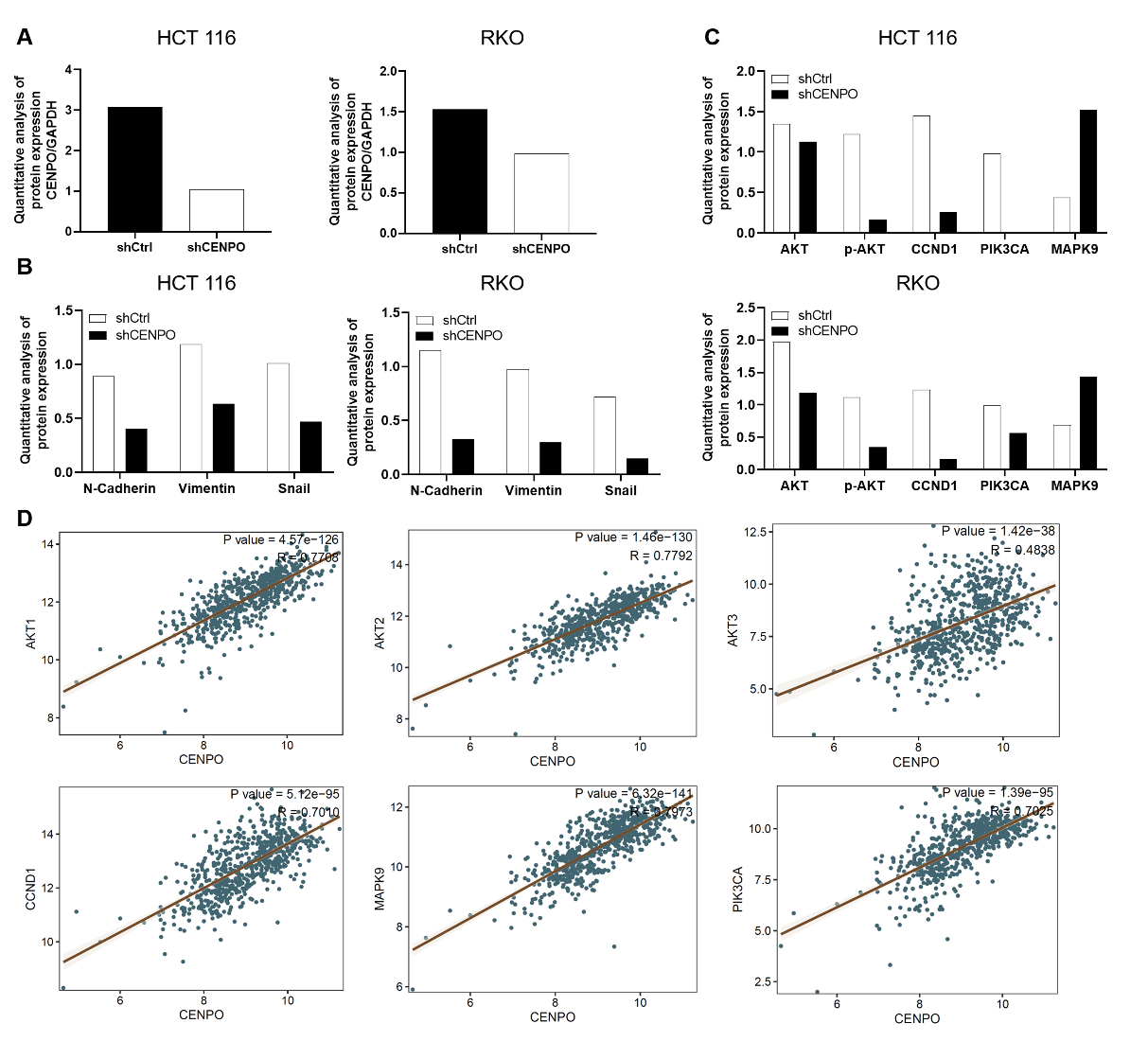


**Fig S2. Quantitative analysis of protein expression results of all western blots.**

(A) Quantification of CENPO protein expression after lentivirus shCtrl and shCENPO interfered with HCT116 and RKO cells. (B) Quantification of EMT-related protein expression in HCT116 and RKO cells by CENPO knockdown. (C) Quantification of the effect of CENPO knockdown on the expression of typical signaling pathway proteins in HCT116 and RKO cells. (D) The correlation between CENPO and AKT, CCND1, PIK3CA and MAPK9 was initially analyzed through Pearson's correlation.

Table S1. The target sequences for the knockdown of CENPO

| Primer Name | Primer Sequence (5’-3’) |
| --- | --- |
| Human-CENPO-1 | AGAAGCATTGGAAGAGAAATT |
| Human-CENPO-2 | GCAGAGAAACCCACTGTGTAA |
| Human-CENPO-3 | CCTGGAAGAGATAGCTGCAAA |

Table S2. Primers used in qPCR

| Primer | Upstream Sequence (5’-3’) | Downstream Sequence (5’-3’) |
| --- | --- | --- |
| CENPO | TGCTTTTGAGGGGAACCTATTG | GGGGAATGAAGACTGGGACT |
| GAPDH | TGACTTCAACAGCGACACCCA | CACCCTGTTGCTGTAGCCAAA |

| Antibody Name | Protein Size (KDa) | Diluted Multiples | Antibody Source | Company | Number |
| --- | --- | --- | --- | --- | --- |
| CENPO | 36 | 1:500 | Rabbit | biorbyt | orb335144 |
| N-cadherin | 125 | 1:1000 | Rabbit | Abcam | ab18203 |
| Vimentin | 54 | 1:2000 | Rabbit | Abcam | ab92547 |
| Snail | 29 | 1:1000 | Rabbit | Abcam | 3879S |
| Akt | 60 | 1:1000 | Rabbit | CST | 4685 |
| p-Akt | 60 | 1:1000 | Rabbit | Bioss | BS-5193R |
| CCND1 | 36 | 1:2000 | Rabbit | CST | 2978 |
| PIK3CA | 110 | 1:1000 | Rabbit | Abcam | ab40776 |
| MAPK9 | 48 | 1:1000 | Rabbit | Abcam | ab76125 |
| GAPDH | 37 | 1:3000 | Rabbit | Bioworld | AP0063 |

Table S3. Antibodies used in western blot
